# Association of *Chlamydia trachomatis* burden with the vaginal microbiota, bacterial vaginosis, and metronidazole treatment

**DOI:** 10.3389/fcimb.2023.1289449

**Published:** 2023-12-12

**Authors:** Caleb M. Ardizzone, Christopher M. Taylor, Evelyn Toh, Rebecca A. Lillis, Jacob H. Elnaggar, John W. Lammons, Patricia Dehon Mott, Emily L. Duffy, Li Shen, Alison J. Quayle

**Affiliations:** ^1^Department of Microbiology, Immunology, and Parasitology, Louisiana State University Health Sciences Center, New Orleans, LA, United States; ^2^Department of Microbiology and Immunology, Indiana University School of Medicine, Indianapolis, IN, United States; ^3^Department of Medicine, Section of Infectious Diseases, Louisiana State University Health Sciences Center, New Orleans, LA, United States

**Keywords:** bacterial vaginosis, *Chlamydia trachomatis*, vaginal microbiome, metronidazole, natural clearance, women, *Lactobacillus*, *Prevotella*

## Abstract

Bacterial vaginosis (BV), a dysbiosis of the vaginal microbiota, is a common coinfection with *Chlamydia trachomatis* (Ct), and BV-associated bacteria (BVAB) and their products have been implicated in aiding Ct evade natural immunity. Here, we determined if a non-optimal vaginal microbiota was associated with a higher genital Ct burden and if metronidazole, a standard treatment for BV, would reduce Ct burden or aid in natural clearance of Ct infection. Cervicovaginal samples were collected from women at enrollment and, if testing positive for Ct infection, at a follow-up visit approximately one week later. Cervical Ct burden was assessed by inclusion forming units (IFU) and Ct genome copy number (GCN), and 16S rRNA gene sequencing was used to determine the composition of the vaginal microbiota. We observed a six-log spectrum of IFU and an eight-log spectrum of GCN in our study participants at their enrollment visit, but BV, as indicated by Amsel’s criteria, Nugent scoring, or VALENCIA community state typing, did not predict infectious and total Ct burden, although IFU : GCN increased with Amsel and Nugent scores and in BV-like community state types. Ct burden was, however, associated with the abundance of bacterial species in the vaginal microbiota, negatively with *Lactobacillus crispatus* and positively with *Prevotella bivia*. Women diagnosed with BV were treated with metronidazole, and Ct burden was significantly reduced in those who resolved BV with treatment. A subset of women naturally cleared Ct infection in the interim, typified by low Ct burden at enrollment and resolution of BV. Abundance of many BVAB decreased, and *Lactobacillus* increased, in response to metronidazole treatment, but no changes in abundances of specific vaginal bacteria were unique to women who spontaneously cleared Ct infection.

## 1 Introduction

*Chlamydia trachomatis* (Ct) serovars D-K are obligate intracellular bacteria that have a unique developmental cycle that typically alternates between an infectious elementary body (EB), a metabolically active but non-infectious reticulate body (RB), and, under stress, a viable but non-cultivable persistent form (Beatty et al., 1994b; Abdelrahman and Belland, 2005; Wyrick, 2010) . The endocervical epithelium is the most common site of Ct infection in women, but bacteria can ascend into the uterus and fallopian tubes, where infection can then lead to severe reproductive sequelae (Brunham and Rey-Ladino, 2005). Infections are predominantly asymptomatic and take months to clear without antibiotic intervention, indicating the ability of the organism to evade the local immune response (Brunham and Rey-Ladino, 2005).

Bacterial vaginosis (BV) is found in approximately 20% to 60% of Ct-infected women, depending on the population demographic (Wiesenfeld et al., 2003; Ziklo et al., 2018; Mott et al., 2021). The vaginal microbiota of healthy women of reproductive age is typically dominated by lactobacilli, whereas BV is characterized by a loss of lactobacilli and overgrowth of opportunistic anaerobic bacteria (Gardner and Dukes, 1955; Hillier, 1993; Hyman et al., 2005; McKinnon et al., 2019; Workowski et al., 2021). BV is generally diagnosed by clinical signs based on Amsel’s criteria and microscopically using Nugent’s scoring system (Amsel et al., 1983; Nugent et al., 1991; McKinnon et al., 2019; Workowski et al., 2021; Colonna and Steelman, 2023), and vaginal bacterial communities can later be classified into community state types by 16S rRNA gene sequencing (McKinnon et al., 2019; France et al., 2020). BV is associated with pelvic inflammatory disease (Haggerty et al., 2016), preterm birth (Hillier et al., 1995), and an increased susceptibility to sexually transmitted infections, including Ct (Wiesenfeld et al., 2003; Bautista et al., 2017). How BV modulates the host-Ct relationship is not fully understood, but it is known that some BV-associated bacteria (BVAB) synthesize indole, a compound which is hypothesized to be utilized by Ct to interfere with interferon gamma (IFNγ)-mediated immunity (Caldwell et al., 2003; Aiyar et al., 2014). Recently, an elevated Nugent score, inclusive of both intermediate microbiota and BV, was reported to be associated with greater chlamydial persistence (Brown et al., 2023), suggesting that disruption of an optimal vaginal microbiota may interfere with Ct clearance. We have also reported that successful metronidazole (MTZ) treatment of BV in Ct-infected women was more likely to permit natural, spontaneous Ct clearance compared to MTZ treatment that failed to resolve BV (Mott et al., 2021). There is also a growing appreciation that the vaginal microbiota and their products modulate multiple aspects of genital innate and adaptive immunity and that there are also functional differences in women with vaginal microbiomes dominated by the beneficial *L. crispatus* compared to the less stable *L. iners* (McKinnon et al., 2019; Armstrong and Kaul, 2021).

Since BV is hypothesized to interfere with anti-chlamydial immunity, we investigated the relationship between optimal and dysbiotic vaginal microbiota and Ct burden and explored how MTZ treatment might affect this relationship. A high Ct burden is associated with upper tract ascension of Ct, which may lead to significant pathology, providing clinical relevance for testing if Ct burden is affected by MTZ treatment of BV (Russell et al., 2016). Further, per current CDC STI treatment guidelines (Workowski et al., 2021), treatment for BV is only recommended for women with symptoms. We reasoned that chlamydial burden should be assessed by both inclusion forming units (IFU) and by total genome copy number (GCN). This is because IFU quantify viable EB that could form additional foci of infection in a host or be transmitted. However, GCN is inclusive of all non-viable and viable forms, including viable but non-replicating Ct forms that are induced in times of environmental stress, which may include those in a BVAB-modulated microenvironment and its induced host immune responses.

Most human Ct research studies are undertaken in women who are returning for treatment of Ct infection after testing positive for Ct by nucleic acid amplification test (NAAT). Since symptomatic BV is usually diagnosed by Amsel’s criteria and treated at the Ct screening visit, we designed a longitudinal clinical study in which women were enrolled, and research samples taken, at the Ct screening visit and again approximately one week later at their Ct treatment visit. This design enabled us to assess the spectrum of Ct burden in a high-risk population prior to antibiotic intervention and determine the contribution of BV and MTZ treatment to Ct burden and clearance.

## 2 Materials and methods

### 2.1 Study population

Women aged 18-35 years who attended the LSU CrescentCare Sexual Health Center in New Orleans, Louisiana were eligible for study recruitment if they were undergoing a Ct and *Neisseria gonorrhoeae* (Ng) NAAT screen and pelvic exam at the enrollment visit (V1) and if they were subsequently found to be positive for Ct infection by NAAT and agreed to a repeat Ct/Ng NAAT and pelvic exam at the Ct treatment and counseling visit (V2). Approval for the study was obtained from the LSUHSC Human Research Ethics Committee, and written informed consent was obtained from each patient. All enrollees completed a detailed questionnaire at both visits. Women who documented that they had oral and/or anal sex were also respectively tested for pharyngeal and/or rectal Ct and Ng. Reasons for exclusion at enrollment were empiric treatment for Ct or Ng and/or antibiotic use in the last 8 weeks. Reasons for exclusion at V1 and V2 were pregnancy or miscarriage in the last 2 months, self-reported sexual intercourse within the last 12 hours, current menstrual bleeding, documented HIV infection or visible HSV lesions. Bacterial vaginosis was assessed in the clinic by Amsel’s criteria.

### 2.2 Collection and processing of clinical samples

Identical sample types were collected at V1 and V2 using our previously described protocols (Mott et al., 2021). Endocervical sampling included a Dacron swab immersed in 1 ml SPG-based Ct transport medium. Cervicovaginal sampling included an Aptima endocervical swab for Ct and Ng testing by the Aptima Combo 2 assay and *Trichomonas vaginalis* (Tv) testing by the Aptima *Trichomonas vaginalis* assay (Hologic, Marlborough, Massachusetts). Vaginal sampling included a Copan swab immediately placed in 1 ml AssayAssure Genelock (Sierra Molecular, Princeton, New Jersey), a cotton swab for pH measurement, whiff test, clue cell counts, and visual Tv assessment, and a cotton swab used for slide preparation for Gram staining for Nugent scoring. Research samples were immediately placed on ice after collection and processed within 2 hours, after which they were stored at -80°C until analysis.

### 2.3 STI testing and treatment

Ct and Ng were detected by the APTIMA Combo 2 test (Hologic, Marlborough, Massachusetts), a target amplification nucleic acid probe test that utilizes target capture for detection of Ct and Ng rRNA. Tv was detected by a wet mount in clinic and confirmed with the APTIMA *Trichomonas vaginalis* assay (Hologic, Marlborough, Massachusetts). BV was diagnosed in the clinic by modified Amsel’s criteria (Amsel score of 3-4 or symptomatic with an Amsel score of 2) and later confirmed by Nugent scoring, and women diagnosed with BV were treated with metronidazole (MTZ) at V1. Women who tested positive by Ct NAAT at V1 were treated with azithromycin or doxycycline at V2, and those with a positive Ng NAAT at V1 were treated with ceftriaxone at V2. Women that developed BV or failed to resolve BV in the interim between V1 and V2 were given MTZ at V2.

### 2.4 Quantification of endocervical *C. trachomatis* burden

Inclusion forming units (IFU) and Ct genome copy number (GCN) were quantified in parallel from the same endocervical swab. IFU were determined as previously described (Ficarra et al., 2008) and imaged and quantified using a BioTek Cytation1 (Agilent, Santa Clara, California). Genomic DNA was isolated from cervical swab eluate using a DNeasy Blood & Tissue Kit (Qiagen, Hilden, Germany), then GCN were determined by quantitative PCR targeting the single chromosomal cytidine triphosphate synthetase (CTPS) gene. Plasmid pET28B cloned with a CTPS gene (kindly provided by A. Aiyar and S. Sherchand) was used as a standard. The PCR setup was as follows: 10 μl SsoAdvanced Universal Probes Supermix 2X (Bio-Rad, Hercules, California), 0.4 μM forward primer (5’-GACAATTGGAGATATAGAATCGCTAC-3’), 0.4 μM reverse primer (5’-CATATGTCATGTGAATGCTAAGGC-3’), 0.2 μM probe (5’-6-FAM/ZEN-TGTACGACAATTCCGCTGCGAACA-Iowa Black FQ-3’), and 3 μl template. All samples were run in duplicate using a CFX96 Real-Time PCR Detection System (Bio-Rad, Hercules, California). Final IFU and GCN were calculated as number per endocervical swab, with a lower limit of quantitation (LLOQ) of 72 IFU/swab and 6,000 GCN/swab.

### 2.5 DNA isolation from vaginal swabs, 16S rRNA gene sequencing, and VALENCIA community state typing

DNA extraction and sequencing were performed at the Indiana University School of Medicine. Genomic DNA was isolated from 400 µl of vaginal swab lysate using the Abbott m2000sp instrument and the mSample Preparation System DNA (Abbott Molecular, Des Plaines, IL), according to instrument set-up prompts and protocol. The instrument script was terminated at the end of the sample processing step, with no further amplification reagents added, and DNA was eluted in 50 µl nuclease-free water. DNA was also extracted from reagent and environmental controls spiked with pure *Thermus thermophilus* HB8 genomic DNA (Takara Bio, San Jose, California) as a reagent contamination control and a ZymoBIOMICS microbial community of known composition (ZymoBIOMICS Microbial Community DNA Standard II, Zymo Research, Irvine, California) as an extraction/annotation control. This mock microbial community contained *Listeria monocytogenes* (89.1%), *Pseudomonas aeruginosa* (8.9%), *Bacillus subtilis* (0.89%), *Saccharomyces cerevisiae* (0.89%), *Escherichia coli* (0.089%), *Salmonella enterica* (0.089%), *Lactobacillus fermentum* (0.0089%), *Enterococcus faecalis* (0.00089%), *Cryptococcus neoformans* (0.00089%), and *Staphylococcus aureus* (0.000089%). 16S sequencing libraries targeting the V4 region of the 16S ribosomal RNA were generated using the NEXTFLEX 16S V4 Amplicon-Seq Library Prep Kit 2.0 (Bioo Scientific, Austin, TX), using a two-step PCR approach as described by the manufacturer. Step one uses 16S gene-specific primer sequences: 515F (5’-GTGCCAGCMGCCGCGGTAA-3’) and 806R (5’-GGACTACHVGGGTWTCTAAT-3’) (Caporaso et al., 2011). In step two, Illumina adaptors and molecular barcoded primer mixes PCRIIF (5’-AATGATACGGCGACCACCGAGATCTACACTCTTTCCCTACACGACGCTCTTCCGATCT-3’) and PCRIIR (5’-CAAGCAGAAGACGGCATACGAGATXXXXXXXXXXXXGTGACTGGAGTTCAGACGTGTGCTCTTCCGATCT-3’) are used to produce the indexed PCR 16S V4 amplicon libraries for sequencing (XXXXXXXXXXXX denotes the index region of the adapter) (Kozich et al., 2013). Mock bacterial community controls (ZymoBIOMICS Microbial Community DNA Standard II, Zymo Research, Irvine, California) and environmental controls spiked with *Thermus thermophilus* HB8 genomic DNA (Takara Bio, San Jose, California) were sequenced in parallel to assess for background signals. Samples were sequenced on an Illumina MiSeq instrument (Illumina, San Diego, California) at the Indiana University Center for Genomics and Bioinformatics, using the version 2 sequencing chemistry (250 bp paired end reads).

FASTQ files were analyzed using R (v4.3.0) with the DADA2 (v1.26.0) and phyloseq (v1.42.0) packages (McMurdie and Holmes, 2013; Callahan et al., 2016; R Core Team, 2022). Raw reads were filtered and trimmed to remove 20 bp from the beginning of each read and 30 bp from the end of each read to remove low quality tails and filter out low quality reads. Error rates were learned, and the DADA algorithm was used to infer forward and reverse sequence variants. These sequence variants were merged and put into a sequence table. Variants outside of the expected range of 273-279 bp were removed from the table and removeBimeraDenovo was used to identify and remove chimeric variants. Taxonomic assignments were classified using the Silva (v138.1) database (Quast et al., 2013). A phylogenetic tree was built using phangorn (v2.11.1) (Schliep, 2011). Potential contaminants and spike in controls were removed using the prevalence method of decontam (v1.18) (Davis et al., 2018). Remaining sequence variants that appeared in only one sample were removed as a prevalence filter and then an abundance filter was applied to remove sequence variants accounting for less than 0.1% on average per sample. The remaining sequence variants were further curated to add species assignments where available using BLAST. The resulting phyloseq object was agglomerated to species level to combine all sequence variants with the same taxonomic classification down to the species level. VALENCIA was used to perform community state type (CST) classification (France et al., 2020), and resulting CST assignments were imported into phyloseq for further analysis.

### 2.6 Statistical analyses

Prior to any statistical analyses, undetectable IFU and GCN in women with a positive Ct NAAT were replaced with half of the LLOQ for each assay. All IFU and GCN were log10-transformed prior to any statistical analyses. Brown-Forsythe and Welch ANOVA was used for comparisons of Ct burden metrics by Amsel score, Nugent score, and community state type. The ANCOM-BC package (v2.2.1) for R was used to assess correlations between Ct burden and vaginal bacterial abundances (Lin and Peddada, 2020). Read counts from all 23 taxa agglomerated to species level were bias-corrected and normalized, then linear modeling was performed using ANCOM-BC. Chlamydial burden metrics were assessed separately as the independent variables in multivariable models following log10-transformation, and race was included as an additional independent variable in all multivariable models assessing the relationship between Ct burden and bacterial abundances. The Holm-Bonferroni method was used to correct for multiple comparisons. Paired t-tests were used for comparisons of Ct burden and relative abundances of vaginal bacteria between enrollment and follow-up visits for each patient (samples missing IFU or GCN for V1 or V2 were excluded from these analyses). All analyses were performed in Graphpad Prism 10 (Dotmatics, Boston, Massachusetts) and R Statistical Software (R Core Team, 2022).

## 3 Results

### 3.1 Characteristics of the patient population

**Figure 1:**
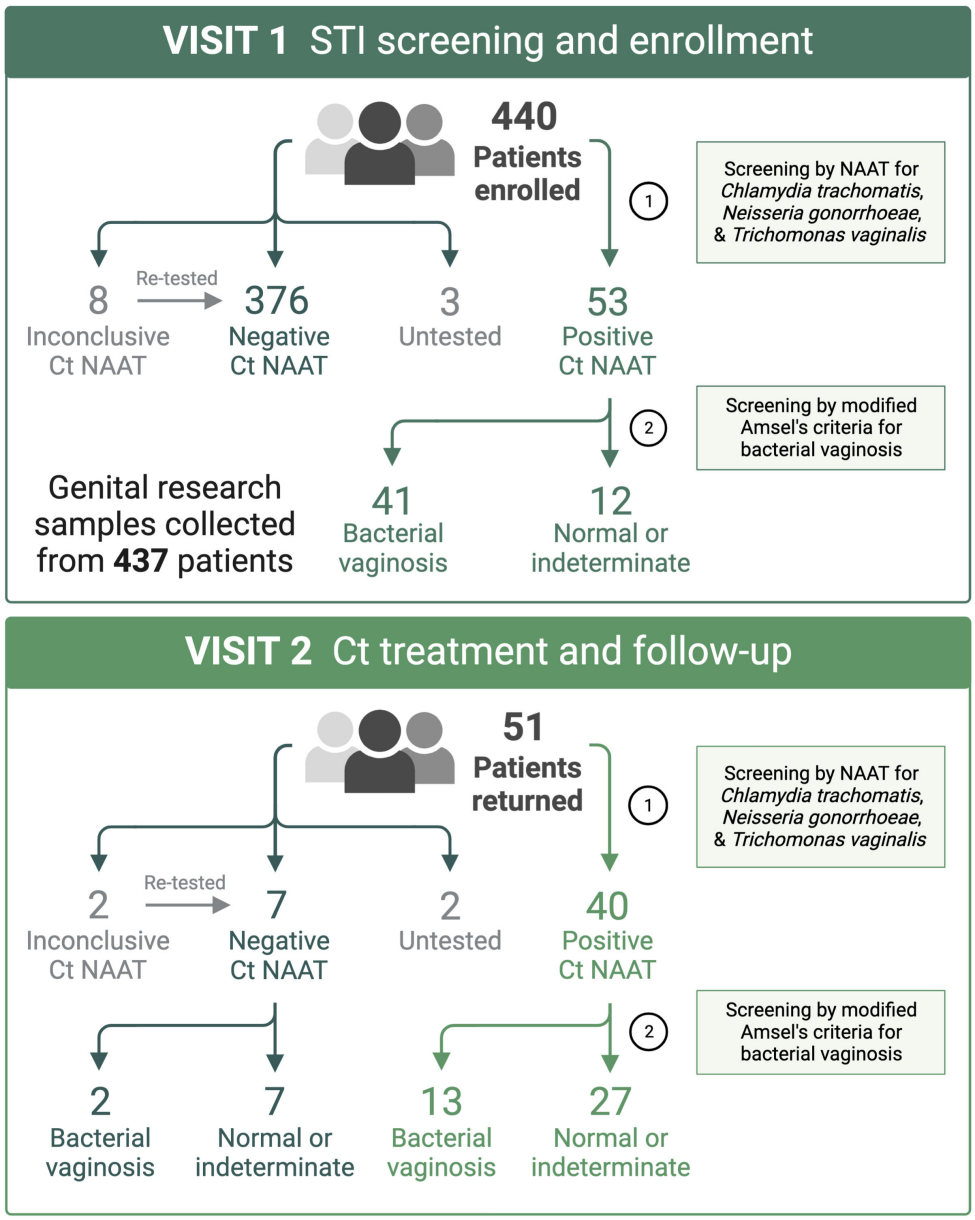
At the STI screening and enrollment visit (V1), BV was diagnosed using Amsel’s criteria by a score of ≥3 or ≥2 when symptomatic. Patients diagnosed with BV were immediately provided a 7-day course of oral metronidazole, apart from two patients that received metronidazole gel. Patients testing positive by Ct NAAT (3 to 4-day turnaround) were immediately scheduled to return to clinic for azithromycin treatment and repeat testing. Created with BioRender.com.

A total of 440 women were enrolled into the study and provided samples at the enrollment visit (V1). Fifty-three (12%) women tested positive by Ct NAAT, 51 of which returned to the clinic for the Ct treatment visit (V2), at which 49 women provided samples and are the focus of this report (**Figure 1**). In women who documented participating in oral or anal intercourse, 3 out of 19 were NAAT-positive for pharyngeal Ct and 4 out of 5 were NAAT-positive for rectal Ct, respectively. Five women were coinfected with *Neisseria gonorrhoeae* (Ng; 10%), and five women were coinfected with *Trichomonas vaginalis* (Tv; 10%), two of which were coinfected with both Ng and Tv (**Supplementary Table 1**). Thirty-nine women (80%) were diagnosed with BV in the clinic using modified Amsel’s criteria, after which women were treated with 7 days of oral metronidazole or 5 days of vaginal metronidazole gel. Women testing positive by Ct NAAT were predominantly young (median 23 years of age, range 18 to 29 years of age), black (80%), and did not use hormonal contraception (61%). The majority of women documented a prior history of STIs (86%), many of which reported prior infection with Ct (80%) or BV (49%).

### 3.2 Spectrum of *Chlamydia* burden at study enrollment

**Figure 2:**
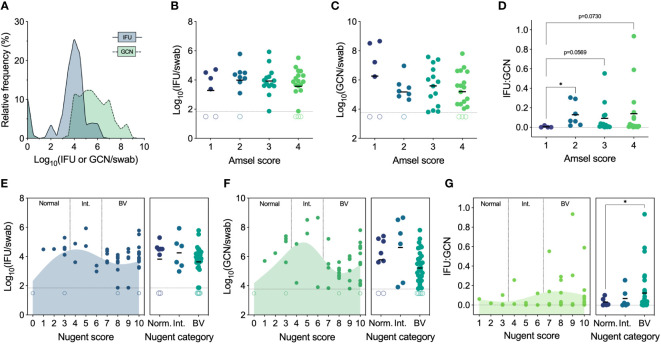
**(A)** Spectrum of chlamydial burden in all patients at the Ct screening and enrollment visit (V1). **(B)** IFU by Amsel score at V1 (n=48). **(C)** GCN by Amsel score at V1 (n=47). **(D)** IFU : GCN by Amsel score at V1 (n=40). **(E)** IFU by Nugent score and category at V1 (n=49). **(F)** GCN by Nugent score and category at V1 (n=48). **(G)** IFU : GCN by Nugent score and category at V1 (n=41). Significance was determined by Brown-Forsythe and Welch ANOVA (*p<0.05).

We first established the full range of endocervical Ct burden found in women who tested positive by Ct NAAT and had not been treated with any antibiotics in the past 3 months. To determine total Ct burden, GCN were determined by quantitative PCR, and IFU were enumerated by culture to quantify infectious Ct burden (i.e., EB). Genome copies are inclusive of all Ct forms, irrespective of viability or infectivity, whereas IFU detect only infectious Ct forms that can be cultured *in vitro*. GCN and IFU were quantified in parallel from the same endocervical swab, revealing a significant spectrum of infectious Ct burden (median 8,282 IFU/swab, IQR 3,453-30,954 IFU/swab, range 0 to 845,279 IFU per swab) and an even greater spectrum of GCN (median 312,040 GCN/swab, IQR 21,743-4,286,604 GCN/swab, range 0 to 453,843,516 GCN per swab) (**Figure 2A**). The proportion of IFU to GCN (IFU : GCN) also varied greatly, with infectious particles constituting the majority of total burden in some individuals and non-infectious particles predominating in others, as has been reported in other studies (Lewis et al., 2014; Mott et al., 2021).

### 3.3 More productive *Chlamydia* infection with a dysbiotic microbiota

Indole-producing BVAB are hypothesized to aid immune escape by Ct, a tryptophan auxotroph, as urogenital strains express a functional tryptophan synthase that enables them to synthesize tryptophan via indole salvage and potentially escape IFNγ-mediated tryptophan depletion (Caldwell et al., 2003; Nelson et al., 2005). We therefore determined if women with a non-optimal vaginal microbiota would have a higher Ct burden than women with an optimal microbiota.

Amsel’s criteria are used in the clinical setting to diagnose BV based on elevated vaginal pH (>4.5), fishy malodor following the addition of 10% potassium hydroxide, thin, grey/white discharge, and presence of clue cells (>20%) (Amsel et al., 1983). Traditionally, an Amsel score of 3 and above is considered indicative of BV, but, in our population, modified Amsel’s criteria are used such that women with an Amsel score of ≥2 and symptomatic for BV were also diagnosed and treated (Workowski et al., 2021). Ct burden, both IFU and GCN, did not differ significantly by Amsel score; however, IFU : GCN ratios did increase with Amsel score (**Figures 2B–D**). No women had an Amsel score of 0 at this first visit, but, compared to an Amsel score of 1, scores of 2 (p=0.0361), 3 (p=0.073), and 4 (p=0.0569) displayed elevated IFU : GCN ratios (**Figure 2D**).

Nugent’s scoring system is considered a gold standard morphological and semi-quantitative evaluation of the vaginal microbiota, evaluating the presence and abundance of large Gram-positive rods (*Lactobacillus*), small Gram-variable rods (*Gardnerella* and *Bacteroides*), and curved Gram-negative rods (*Mobiluncus*) (Nugent et al., 1991). A score of 0-3 is considered normal, 4-6 is considered indeterminate for BV (i.e., intermediate), and 7-10 is considered indicative of BV. IFU and GCN did not differ significantly when assessed by Nugent score or category (**Figures 2E, F**). However, both IFU and GCN followed similar trends, increasing from a score of 0 (normal) up to a score of 5 (intermediate), decreasing approaching a score of 8 (BV), then increasing approaching the maximum score of 10 (**Figures 2E, F**). IFU : GCN ratios were higher in patients with Nugent scores of 7-10 compared to 0-3 (p=0.0401; **Figure 2G**). Taken together, these results suggest that BV status, by either Amsel’s criteria or Nugent’s scoring system, does not strongly predict infectious or total Ct burden. We also noted higher IFU : GCN ratios in BV, possibly indicative of a more productive Ct infection. However, high IFU : GCN ratios can be reached through increased IFU, decreased GCN, or a mix of both, complicating the interpretation of these results.

### 3.4 Total, but not infectious, *Chlamydia* burden differs by community state type

BV can vary greatly in its composition, typically involving a multitude of bacterial species. Given that increased IFU : GCN ratios were primarily observed in a subset of women with BV, we reasoned the presence and abundance of specific bacterial species present in this subset of cases may explain this divergence. To better characterize the vaginal microbiota of each patient, 16S rRNA gene sequencing was performed, and samples were assigned to community state types based on their taxonomic composition using VALENCIA, a nearest-centroid classifier that categorizes samples into five major discrete community state types using ASVs (France et al., 2020). While operational taxonomic units (OTUs) have been used historically to assess microbial communities, microbiome studies have generally moved to ASV as a more accurate method of assessment for 16S rRNA gene sequencing reads in the seven years since DADA2 has been published (Callahan et al., 2016). OTU clustering was a computational efficiency that generally loses resolution of reads whereas ASV analysis maintains repeatable biological variation, and this additional resolution provided by ASVs allows for more reads to be classified to more specific levels of taxonomy (Callahan et al., 2017).

**Figure 3:**
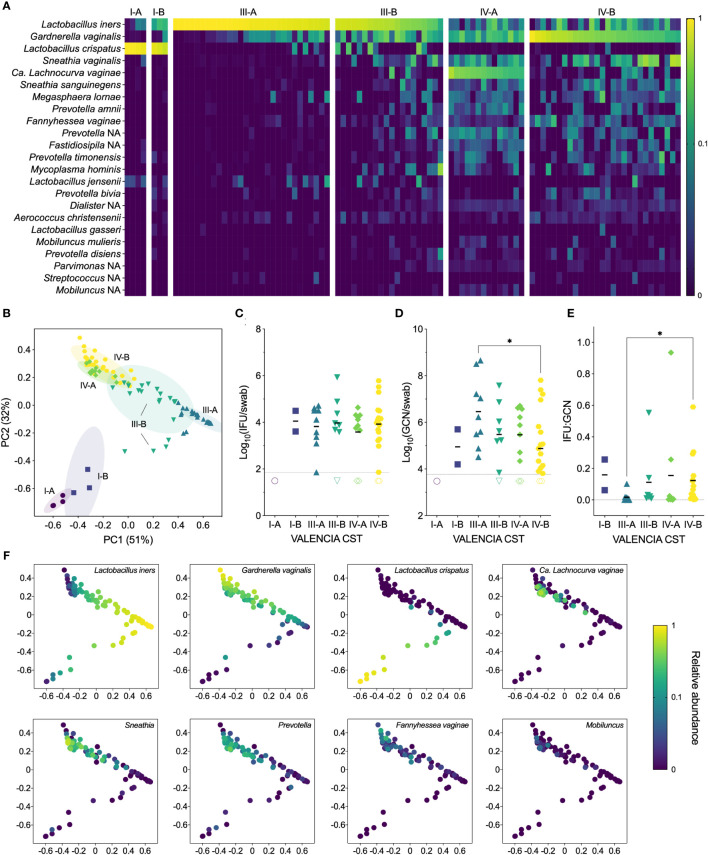
**(A)** Heatmap of the relative abundances of vaginal bacterial taxa identified by 16S rRNA gene sequencing, showing both clinical visits (n=98). **(B)** Ordination plot of the vaginal microbiome, grouped with VALENCIA-assigned CSTs, showing both clinical visits (n=98). **(C)** IFU by VALENCIA CST at V1 (n=49). **(D)** GCN by VALENCIA CST at V1 (n=48). **(E)** IFU : GCN by VALENCIA CST at V1 (n=41). **(F)** Ordination plot colored by abundance of dominant taxa: *Lactobacillus iners*, *Gardnerella vaginalis*, *Lactobacillus crispatus*, *Candidatus Lachnocurva vaginae*, *Sneathia*, *Prevotella*, *Fannyhessea vaginae*, and *Mobiluncus* (n=98). Significance was determined by Brown-Forsythe and Welch ANOVA (*p<0.05).

Samples from our cohort fell into three state types: CST I, dominated by *L. crispatus*, CST III, dominated by *L. iners*, and CST IV, characterized by a diverse array of BV-associated anaerobes (**Figures 3A, B**). Each CST was then divided into sub-CSTs, where possible. CST I was split into CST I-A, with clear *L. crispatus* dominance (median 96%, range 88-98%), and CST I-B, with a lower relative abundance of *L. crispatus* (median 76%, range 58-83%). CST III was split into CST III-A, which was characterized by a high relative abundance of *L. iners* (median 94%, range 59-100%), and CST III-B, which had a somewhat lower relative abundance of *L. iners* (median 50%, range 16-73%). For both CST I and CST III, the ‘A’ subsets tended to have very high relative abundances of the dominant *Lactobacillus* species. (i.e., >90%), although some women had mixed communities of lactobacilli, including *L. jensenii* and *L. gasseri*. The ‘B’ subsets tended to have lower abundances of the dominant *Lactobacillus* species. and include more BV-associated anaerobes. For this reason, these “B” subsets often presented as BV in the clinic, particularly for CST III-B, of which 85% were diagnosed with BV and treated. CST IV was divided into CST IV-A, characterized by a high abundance of *Candidatus Lachnocurva vaginae*, formerly BVAB1 (Holm et al., 2020), with some *Gardnerella vaginalis*, and CST IV-B, characterized by a high abundance of *G. vaginalis* with some *Ca. L. vaginae*. Nearly all women classified as CST IV were diagnosed with BV and subsequently treated with metronidazole (98%). However, only 72% of clinically diagnosed cases of BV were classified as CST IV, with the remaining BV cases classified as CST III-B (16%), CST III-A (9%), and CST I-B (3%).

Next, we compared chlamydial burden between VALENCIA-assigned sub-CSTs. As with Amsel and Nugent scores, infectious Ct burden did not significantly differ between any sub-CSTs, although GCN appeared to be highest in CST III-A, which significantly differed from CST IV-B (**Figures 3C, D**). The IFU : GCN ratios of CST III-A were significantly lower than those of CST IV-B (p=0.0263), mostly attributable to the increased GCN (**Figure 3E**). Taken together, these results indicate that CST classifications are poorly predictive of infectious and total Ct burden. However, differences in IFU : GCN ratios may suggest alteration of chlamydial growth states by vaginal microbial communities. Additionally, variance in relative abundances of vaginal bacterial species not only between but within CSTs further complicates interpretation of these results (**Figure 3F**), prompting us to examine the relationship between Ct burden and individual bacterial species rather than community-level classifications.

### 3.5 *Chlamydia* burden negatively associates with *Lactobacillus crispatus* abundance and positively associates with *Prevotella bivia*

**Figure 4:**
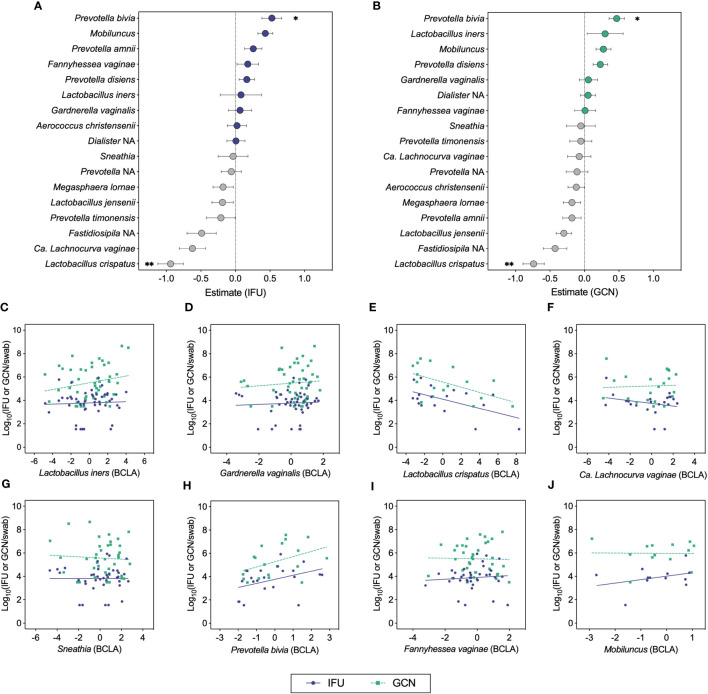
ANCOM-BC analyses correlating chlamydial burden with bias-corrected log-abundances (BCLA) of vaginal bacterial taxa. **(A, B)** ANCOM-BC model estimates representing correlations between vaginal bacterial taxa and IFU (n=49) or GCN (n=48), respectively. **(C–J)** BCLA of prominent vaginal taxa against IFU (blue circles, solid line) and GCN (green squares, dashed line). Significance was determined by linear modeling in ANCOM-BC, and the Holm-Bonferroni method was used to correct for multiple comparisons (*q<0.05, **q<0.01).

We next used ANCOM-BC to test the relationship between total and infectious Ct burden and the bias-corrected log-abundances of vaginal bacterial genera and species (**Figures 4A–J**) (Lin and Peddada, 2020). This method revealed inverse associations between both IFU and GCN with *L. crispatus* and positive associations with *P. bivia* (**Figures 4A, B, E, H**). Most of the samples with detectable *L. crispatus* corresponded to mixed bacterial communities in which *L. crispatus* was not the dominant *Lactobacillus* species, yet the contribution of *L. crispatus* in these communities was sufficient to observe a negative effect on chlamydial growth. Negative associations were also apparent for *L. jensenii* with both IFU and GCN, displaying trends similar to those of the *L. crispatus*. Weaker associations were visible for other BV-associated bacteria, with IFU trending towards a negative association with *Ca. L. vaginae* (q=0.066; **Figure 4F**) and *Mobiluncus* (q=0.0574; **Figure 4I**). Using this method, IFU : GCN ratios did not associate significantly with any bacterial taxa other than a single positive association with *P. amnii* (q=0.003; **Supplementary Figure 1**). No significant association was identified between Ct burden and *L. iners* abundance, suggesting that other bacteria in these communities may be of greater importance in modulating chlamydial survival and growth. Taken together, these results suggest that chlamydial survival and/or growth may be modulated by specific vaginal bacteria and their associated microenvironments, most notably by *L. crispatus* and *Prevotella*.

### 3.6 Spontaneous clearance of *Chlamydia* associates with successful MTZ treatment and a low chlamydial burden at screening

**Figure 5:**
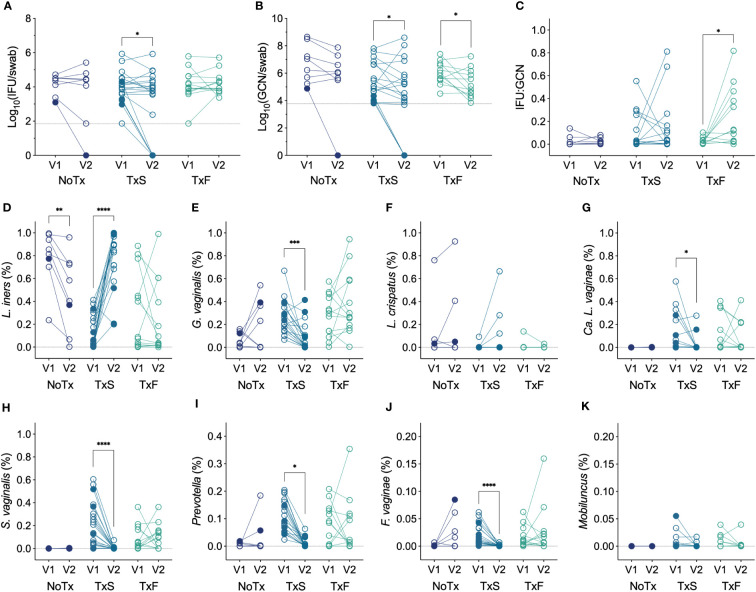
**(A)** Changes in IFU from V1 to V2 by modified Amsel’s criteria and MTZ treatment status (n=41). **(B)** Changes in GCN from V1 to V2 by modified Amsel’s criteria and MTZ treatment status (n=39). **(C)** Changes in IFU : GCN from V1 to V2 by modified Amsel’s criteria and MTZ treatment status (n=33). **(D–K)** Changes in relative abundance of prominent vaginal taxa from V1 to V2 by MTZ treatment status (n=41). Filled circles represent women that naturally cleared Ct infection, and hollow circles indicate non-clearers. NoTx, no treatment; TxS, treatment success; TxF, treatment failure. Significance between V1 and V2 within each treatment group was determined by Wilcoxon matched-pairs signed rank tests (*p<0.05, **p<0.01, ***p<0.001, ****p<0.0001).

We next evaluated whether Ct burden responded to significant changes in the vaginal microbiota and, specifically, the MTZ-mediated resolution of BV using paired specimens from the enrollment and follow-up visits (V1 and V2). Since oral MTZ treatment is unsuccessful in a significant proportion of women (35%) (Larsson and Forsum, 2005; Marrazzo et al., 2008), we divided patients by treatment success or failure using modified Amsel’s criteria, and also compared these groups with women who were not treated with MTZ. In patients with detectable chlamydial burden at V1, both total and infectious chlamydial burden significantly decreased between V1 and V2 in women that resolved symptomatic BV following MTZ treatment (**Figures 5A, B**), and IFU : GCN ratios significantly increased in cases of MTZ treatment failure (**Figure 5C**). However, these differences in Ct burden were mainly attributable to a group of four women who spontaneously cleared chlamydial infection by V2 (negative Ct NAAT) and one woman with undetectable IFU and GCN but a positive Ct NAAT at V2. Also, a small but significant reduction in GCN, but not IFU, was observed in non-clearers treated with MTZ, regardless of whether the treatment was successful (**Supplementary Figures 2A, B**), and this was accompanied by a significant increase in IFU : GCN (**Supplementary Figure 2C**), suggesting that MTZ treatment may aid in reducing total Ct burden even when BV is not resolved. Every Ct clearer but one had less than 2,000 IFU per swab (cohort median 8,282, IQR 3,453-30,954) and 22,000 GCN per swab (cohort median 312,040, IQR 21,743-4,286,604) at V1, which was significantly less than that of non-clearers (**Supplementary Figures 3A, B**). In Ct clearers identified by Ct NAAT but who lacked detectable Ct burden at V1, one had a *L. crispatus*-dominant microbiota and did not require MTZ treatment (Amsel 1, Nugent 0, CST I-A), two successfully resolved BV after MTZ treatment, and one failed to resolve BV after MTZ treatment. These results suggest that MTZ treatment and resolution of BV may aid in the natural, spontaneous clearance of chlamydial infection, particularly in cases of low chlamydial burden.

Given the innate diversity of BV, differing susceptibilities of BVAB to MTZ (Ferris et al., 2007; Armstrong et al., 2022; Gustin et al., 2022), and previous significant correlations of vaginal bacteria with chlamydial burden, we assessed changes in prominent vaginal bacterial species in the same groups of patients (MTZ treatment success, MTZ treatment failure, and no treatment) and looked for trends unique to women that naturally cleared Ct infection. We observed that women successfully treated with MTZ had increases in the relative abundances of *L. iners* or *L. crispatus* and reductions in the those of many BVAB, such as *G. vaginalis*, *Ca. L. vaginae*, *S. vaginalis*, *F. vaginae*, and *Prevotella*, as would be expected (**Figures 5D–I**). A decrease in *L. iners* was observed in women that were not treated with MTZ (**Figure 5D**), consistent with the instability of *L. iners*-dominant communities (van de Wijgert et al., 2014; McKinnon et al., 2019; Carter et al., 2023). Given the low number of patients with detectable Ct burden at V1 that cleared Ct infection by V2, we were not able to detect unique, significant changes in the abundances of specific vaginal bacteria of clearers compared to non-clearers, and we did not observe any trends to suggest that clearers differ from non-clearers in this way.

## 4 Discussion

Ct infections are typically chronic, and reinfection is common, indicating that immunity in many women may only be partially protective or compromised. A higher Ct burden is associated with transmission and ascension, and chronic upper tract infection is associated with pelvic inflammatory disease (Brunham and Rey-Ladino, 2005; Russell et al., 2016; Schillinger et al., 2016). Determining the endogenous and exogenous factors that contribute to the intractability and magnitude of chlamydial burden will be helpful for developing new strategies to reduce both individual and population-based Ct burden.

Clinical diagnoses of chlamydial infections are made using highly sensitive NAATs that detect extremely small amounts of Ct nucleic acid (e.g., <1 IFU/assay or <7.25 IFU/swab by the Aptima Combo 2 NAAT) and do not require viable organisms. Results are reported as positive, negative, or indeterminate with no indication of the extent of the Ct infection. Many current research studies quantify only total burden, but this does not inform if bacteria are viable, replication competent, or infectious and could therefore amplify infection within a host or be transmitted to a sexual partner. In contrast, parallel evaluation of IFU and GCN informs both infectious and total burden, and IFU : GCN ratios inform the proportion of bacteria that are infectious at the time of sampling (Lewis et al., 2014). This is important, as *in vitro* studies indicate that the growth characteristics of Ct, an obligate intracellular pathogen with a small genome size and multiple auxotrophies, are driven by environmental conditions. Under stressful conditions *in vitro*, such as generic depletion of amino acids, limitation of tryptophan, isoleucine, glucose, or iron, and the presence of viral coinfections or certain antibiotics, Ct enters into a persistent, viable, non-infectious form which may or may not be able to re-enter the developmental cycle, depending on the magnitude and duration of stress (Coles et al., 1993; Beatty et al., 1994a; Beatty et al., 1994b; Harper et al., 2000; Belland et al., 2003; Hogan et al., 2004; Leonhardt et al., 2007; Hatch and Ouellette, 2020; Riffaud et al., 2023). Persistent forms are also differentially sensitive to antibiotics (Reveneau et al., 2005; Marangoni et al., 2020) and may aid in establishing long-term intractable reservoirs of infection (Dean et al., 2000; Suchland et al., 2017). Here, we illustrated the immense spectrum of infectious chlamydial burden and even broader spectrum of total chlamydial burden in untreated infection and how IFU : GCN reveals a heterogeneity of Ct growth with some samples predominated by replicating forms, and others by non-replicating forms, supporting our previous pilot study (Lewis et al., 2014). However, our future studies would benefit from the use of a recently described Ct-specific viability-PCR capable of distinguishing viable from non-viable Ct (Janssen et al., 2016; Janssen et al., 2018; Dukers-Muijrers et al., 2020; Janssen et al., 2020; Versteeg et al., 2020; Janssen et al., 2022; Vojtech et al., 2023).

BV is a heterogeneous condition with various, and evolving, classifications (McKinnon et al., 2019). Multiple lines of evidence indicate, however, that the molecular composition of the genital environment is significantly modulated by anaerobe proliferation, inter-microbe interactions, and the ensuing host response (Mitchell and Marrazzo, 2014; Schaffer and Taylor, 2015; Srinivasan et al., 2015; Schirmer et al., 2016; Oliver et al., 2020). Based on the indole immune-escape hypothesis (Caldwell et al., 2003; Nelson et al., 2005), we expected that women with untreated BV might have a greater Ct burden than those with *Lactobacillus*-dominant microbiota, reasoning that BV would hinder eradication and promote spread of the Ct. However, we found no significant difference in IFU and GCN with respect to Amsel score, Nugent category, or VALENCIA-assigned CST, indicating that community-level classifications of the vaginal microbiota alone are not predictive of infectious or total chlamydial burden. There were, however, clear differences in IFU : GCN ratios between vaginal bacterial communities, potentially representing a spectrum of Ct growth states. Regardless of whether Amsel scores, Nugent categories, or CST were used, IFU : GCN ratios were elevated in women with BV, suggesting that this environment may promote the most productive Ct growth. However, IFU : GCN ratios are only one of many parameters that have been used to contrast productive and persistent growth states, and other confirmatory approaches, such as morphological and transcriptional characterization of Ct forms, are required to more confidently differentiate Ct growth states (Gérard et al., 2002; Belland et al., 2003; Gérard et al., 2004; Lewis et al., 2014). When individual bacterial species within these communities were examined, *L. crispatus* abundance was found to inversely correlate with both total and infectious chlamydial burden, consistent with published studies describing the partial protective effect of *L. crispatus* on Ct infection, potentially via the production of lactic acid and other metabolites or by modulation of inflammation in the genital tract (O'Hanlon et al., 2013; Gong et al., 2014; Nardini et al., 2016; Anton et al., 2018; Edwards et al., 2019; Rastogi and Singh, 2022; Dong et al., 2023; Zalambani et al., 2023). Additionally, both total and infectious chlamydial burden positively correlated with the abundance of *P. bivia*, and IFU : GCN ratios with *P. amnii*. However, not all *Prevotella* species followed this trend. While members of the genus *Prevotella* are known to express tryptophanase, an enzyme capable of catabolizing tryptophan to indole, little is known about if and which specific *Prevotella* species in the vaginal microbiota possess this capability, which is further complicated by variation in indole production between different isolates of the same species (Könönen et al., 1998; Lawson et al., 2008; Sasaki-Imamura et al., 2011; Ziklo et al., 2016). While our 16S rRNA gene sequencing data is unable to address this specific knowledge gap, our planned studies will be addressing this with shotgun metagenomic sequencing.

Natural, spontaneous clearance of chlamydial infection in the short interval between screening and treatment has been documented by us and others (Geisler et al., 2008; Geisler et al., 2013; Mott et al., 2021). Individuals who naturally clear infection and are protected from reinfection are important to study as they can help define the correlates of immunity to Ct (Geisler et al., 2008; Geisler et al., 2013). Using the current ‘screen to treat’ definition of a natural clearer, namely Ct NAAT-positive at V1 and Ct NAAT-negative at V2, we observed a clearance rate similar to previous studies (17%) (Geisler et al., 2008; Geisler et al., 2013; Mott et al., 2021). However, using our in-house assays for determining IFU and GCN, we noted that three samples from these clearers had values below the LLOQ of these assays at V1. For the longitudinal analyses, we modified our definition of clearance to include women with detectable IFU and GCN at V1 and undetectable IFU and GCN at V2, identifying three additional women. Utilizing these individuals, we made several observations, namely that all but one of the identified clearers were successfully treated with MTZ and that Ct burden in these women was significantly lower than women who did not clear infection, even within the same group. This low Ct burden at V1 would suggest that an appropriate local adaptive immune response had been generated, only needing the ‘BV break’ to fully resolve the infection. It is important to note that clinical diagnoses of BV by Amsel’s criteria do not always agree with the results of Nugent’s scoring system and community state typing. However, we chose to use modified Amsel’s criteria to evaluate treatment success, given that these criteria were used to diagnose and treat BV at V1. These findings also illustrate that, when investigating the relationship between Ct burden, Ct clearance, and the genital milieu, it may be most informative to focus on samples with documented, significant changes in Ct burden, rather than ‘screen to treat’ clearance where Ct burden is most often minimal. It is important to note that this was an opportunistic, exploratory investigation utilizing a subset of samples that were collected as a part of a larger study using unrelated statistical power and sample size estimates. While we did not detect statistically significant differences in some comparisons, this may be due to the low number of patients included in this exploratory analysis, particularly natural clearers with detectable Ct burden at enrollment.

Why might successful, but not unsuccessful, MTZ treatment of women aid in clearance of low burden Ct infection in some women? There may be several, unlikely independent, explanations. Successful MTZ treatment typically reduces the burden of many BVAB, including indole producing *Prevotella* (Srinivasan et al., 2015; Armstrong et al., 2022). The MTZ-mediated reduction in some BVAB may also reduce proteases and sialidases responsible for cleaving and abrogating antibody function or mucinases, which alter the integrity and function of the mucus barrier (Cauci et al., 1998; Lacroix et al., 2020). MTZ either directly, or indirectly through bacterial burden reduction and subsequent environmental changes, also modulates many proinflammatory cytokines and chemokines (Yudin et al., 2003; Roth et al., 2010; Serebrenik et al., 2021; Armstrong et al., 2022). Some of these changes in conditions may favor Ct clearance, such as the documented increase in IP-10, which may attract T cells to the site of infection (Mott et al., 2021; Serebrenik et al., 2021; Armstrong et al., 2022). In this study, while the use of relative abundances was sufficient to detect significant reductions of many BVAB and increases in *Lactobacillus* following successful MTZ treatment, the magnitude of the effects that these bacteria have on the genital environment are directly linked to their absolute abundances or burden, the quantification of which may be required to understand the relationship between the vaginal microbiota, genital environment, and chlamydial clearance. Future studies would benefit from the quantification of absolute abundances of dominant vaginal bacterial taxa or by inferring absolute abundances from total bacterial burden in combination with compositional data (Tettamanti Boshier et al., 2020; Elnaggar et al., 2023a; Elnaggar et al., 2023b).

Clearly, the interactions between the genital microbiota and Ct are more complex to evaluate than originally anticipated. However, a new model may be emerging from ours and others recent data (Geisler et al., 2008; Geisler et al., 2013; Mott et al., 2021; Brown et al., 2023; Jordan et al., 2023), and our rapidly increasing understanding of the microbiota at this unique site. First, given the maintenance of tryptophan synthase in nearly all genital Ct isolates, there is little reason to doubt that the net presence of indole is an important mechanism for Ct escape (Caldwell et al., 2003; Nelson et al., 2005; Ziklo et al., 2016; Sherchand and Aiyar, 2019; Mott et al., 2021). However, this is also possible in women who are classified with intermediate microbiota in addition to those with BV (France et al., 2020). Second, emerging vaginal metabolome data indicate that BVAB deplete amino acids (Srinivasan et al., 2015), which may create a less-than-optimal environment for Ct replication and survival (Riffaud et al., 2023). This may provide one explanation of the recent data in which Ct clearers had less robust amino acid profiles than non-clearing individuals matched by BV status as indicated by Nugent scoring (Jordan et al., 2023). Third, many metabolic products of BVAB aside from indole, including additional tryptophan catabolites, can directly modulate Ct growth cues or local immunity (Zelante et al., 2014; Cella and Colonna, 2015; Gao et al., 2018; Sherchand and Aiyar, 2019). It is possible that Ct survival and replication is optimal in a mildly dysbiotic community dominated by *L. iners* since this environment allows for the growth of anaerobes beneficial to Ct growth without the byproducts, inflammation, and hypoxia of severe BV. Further studies are needed to elucidate these complex relationships, and these studies may benefit from the incorporation of longitudinal protocols that allow for morphological and molecular characterization of Ct forms in parallel with comprehensive assessments of metabolomic and host immune parameters in order to understand which and how genital environmental factors and stressors modulate Ct replication and survival.

## Data availability statement

The datasets presented in this study can be found in online repositories. The names of the repository/repositories and accession number(s) can be found below: https://www.ncbi.nlm.nih.gov/, BioProject ID PRJNA1013329.

## Ethics statement

The studies involving humans were approved by LSUHSC Human Research Ethics Committee. The studies were conducted in accordance with the local legislation and institutional requirements. The participants provided their written informed consent to participate in this study.

## Author contributions

CA: Conceptualization, Data curation, Formal analysis, Investigation, Methodology, Visualization, Writing – original draft, Writing – review & editing, Supervision, Validation. CT: Conceptualization, Data curation, Formal analysis, Funding acquisition, Investigation, Methodology, Project administration, Software, Supervision, Validation, Writing – review & editing. ET: Methodology, Writing – review & editing. RL: Conceptualization, Funding acquisition, Investigation, Methodology, Project administration, Supervision, Writing – review & editing. JE: Investigation, Validation, Writing – review & editing. JL: Formal analysis, Methodology, Software, Writing – review & editing. PD: Data curation, Investigation, Methodology, Writing – review & editing. ED: Investigation, Writing – review & editing. LS: Resources, Writing – review & editing. AQ: Conceptualization, Funding acquisition, Project administration, Supervision, Validation, Writing – original draft, Writing – review & editing.
