# Supplementary material for: Association of Chlamydia trachomatis burden with the vaginal microbiota, bacterial vaginosis, and metronidazole treatment
Source: Front Cell Infect Microbiol. 2023 Dec 12;13:1289449. doi: 10.3389/fcimb.2023.1289449 (PMC10750252; doi:10.3389/fcimb.2023.1289449)
Supplement: Supplementary file 1 [file DataSheet_1.docx]

| **Characteristic** | **Total (n=49)** |
| --- | --- |
| Age, median (range) | 23 (18-29) |
| Race |  |
| Black | 43 (88%) |
| White | 5 (10%) |
| Asian | 1 (2%) |
| Contraception |  |
| Hormonal | 17 (35%) |
| None | 30 (61%) |
| Other | 2 (4%) |
| STI history |  |
| Any prior STI | 42 (86%) |
| *Chlamydia trachomatis* | 39 (80%) |
| *Neisseria gonorrhoeae* | 12 (24%) |
| *Trichomonas vaginalis* | 11 (22%) |
| Bacterial vaginosis | 24 (49%) |
| *C. trachomatis* NAAT |  |
| Vaginal/endocervical | 49 (100%) |
| Oropharyngeal | 3 (6%) |
| Rectal | 4 (8%) |
| Coinfections |  |
| *Neisseria gonorrhoeae* | 5 (10%) |
| *Trichomonas vaginalis* | 5 (10%) |
| Bacterial vaginosis | 39 (80%) |
| Amsel's criteria scores |  |
| Incomplete criteria | 1 (2%) |
| Total score 0-1 | 6 (12%) |
| Total score 2 | 9 (18%) |
| Total score 3 | 15 (31%) |
| Total score 4 | 18 (37%) |

**Supplementary Table 1.** Characteristics of study participants with a positive *C. trachomatis* NAAT result. Data are reported as number (percent) unless specified otherwise. Only women who documented that they had oral and/or anal sex were tested for oropharyngeal and/or rectal Ct and Ng, respectively. One woman without BV lacked complete Amsel’s criteria and was not assigned an Amsel score. Of the nine women with an Amsel score of 2, six with vaginitis symptoms were diagnosed with BV. Abbreviations: STI, sexually transmitted infection; NAAT, nucleic acid amplification test.

**
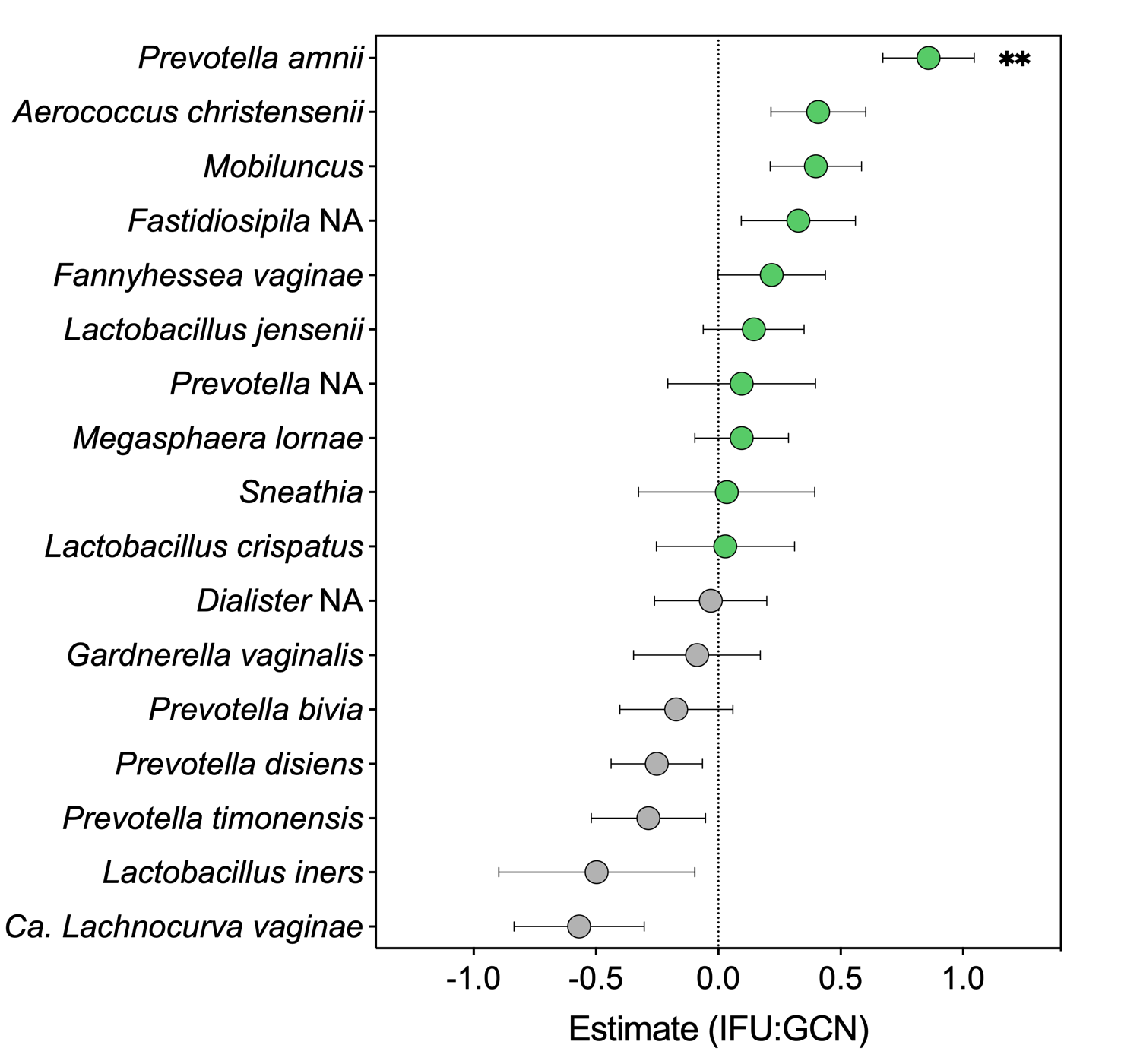
**

**Supplementary Figure 1.** ANCOM-BC model estimates representing correlations between vaginal bacterial taxa and IFU:GCN (n=49). Significance was determined by linear modeling in ANCOM-BC, and the Holm-Bonferroni method was used to correct for multiple comparisons (*q<0.05, **q<0.01).

**
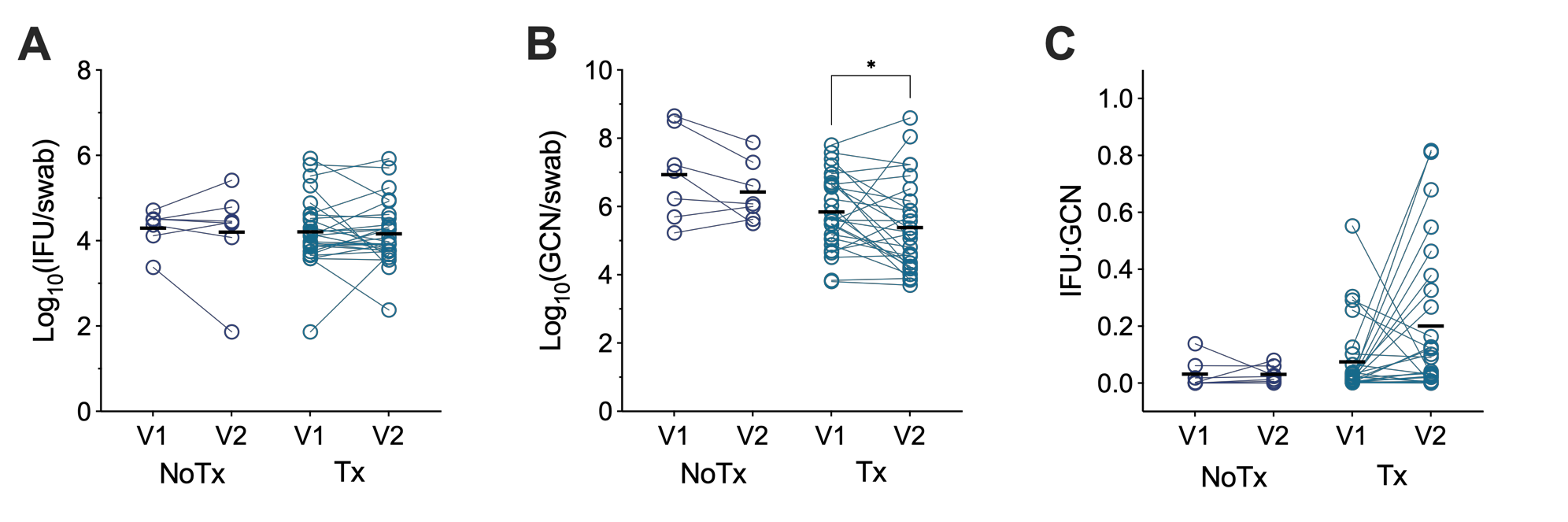
**

**Supplementary Figure 2.** (**A**) Changes in IFU of non-clearers from V1 to V2 by MTZ treatment status (n=35). (**B**) Changes in GCN of non-clearers from V1 to V2 by MTZ treatment status (n=34). (**C**) Changes in IFU:GCN of non-clearers from V1 to V2 by MTZ treatment status (n=33). Abbreviations: no treatment (NoTx), treatment (Tx). Significance determine by Wilcoxon matched-pairs signed rank test (*p<0.05).


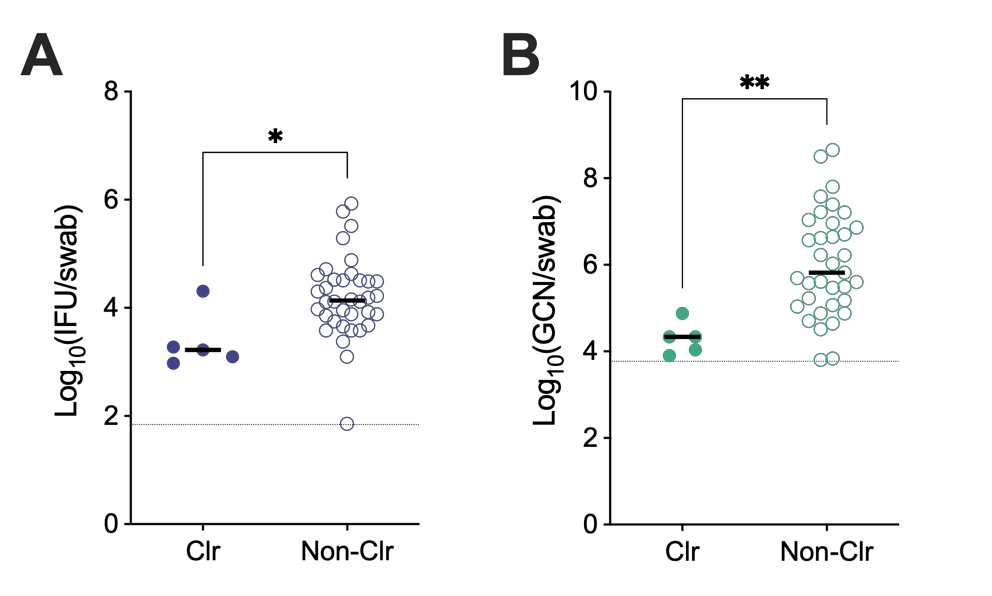


**Supplementary Figure 3.** (**A**) IFU of clearers vs non-clearers at V1 (n=41). (**B**) GCN of clearers vs non-clearers at V1 (n=40). Patients without detectable burden at V1 were not included in this comparison. Significance determined by Mann-Whitney test (*p<0.05, **p<0.01).
